# Dynamic regulation of airway surface liquid pH by TMEM16A and SLC26A4 in cystic fibrosis nasal epithelia with rare mutations

**DOI:** 10.1073/pnas.2307551120

**Published:** 2023-11-15

**Authors:** Livia Delpiano, Lisa W. Rodenburg, Matthew Burke, Glyn Nelson, Gimano D. Amatngalim, Jeffrey M. Beekman, Michael A. Gray

**Affiliations:** ^a^Biosciences Institute, Faculty of Medical Sciences, Newcastle University, Newcastle upon Tyne NE2 4HH, United Kingdom; ^b^Department of Pediatric Pulmonology, Wilhelmina Children’s Hospital, University Medical Center Utrecht, Utrecht University, Member of the European Reference Network-LUNG, Utrecht 3584 EA, The Netherlands; ^c^Regenerative Medicine Center Utrecht, University Medical Center Utrecht, Utrecht University, Utrecht 3584 CT, The Netherlands; ^d^Bioimaging Unit, Ageing Research Laboratories, Campus for Ageing and Vitality, Newcastle University, Newcastle Upon Tyne NE4 5PL, United Kingdom; ^e^Centre for Living Technologies, Alliance Eindhoven University of Technology, Wageningen University and Research, Utrecht University, University Medical Center Utrecht, Utrecht 3584 CB, The Netherlands

**Keywords:** cystic fibrosis, airway surface liquid pH, inflammation, SLC26A4, TMEM16A

## Abstract

In cystic fibrosis (CF), defects in the CF transmembrane conductance regulator (CFTR) channel lead to an acidic airway surface liquid (ASL), which compromises innate defence mechanisms, predisposing to pulmonary failure. Restoring ASL pH is a potential therapy for people with CF, particularly for those who cannot benefit from current highly effective modulator therapy. However, we lack a comprehensive understanding of the complex mechanisms underlying ASL pH regulation. The calcium-activated chloride channel, TMEM16A, and the anion exchanger, SLC26A4, have been proposed as targets for restoring ASL pH, but current results are contradictory and often utilise nonphysiological conditions. To provide better evidence for a role of these two proteins in ASL pH homeostasis, we developed an efficient CRISPR-Cas9-based approach to knock-out (KO) relevant transporters in primary airway basal cells lacking CFTR and then measured dynamic changes in ASL pH under thin-film conditions in fully differentiated airway cultures, which better simulate the in vivo situation. Unexpectantly, we found that both proteins regulated steady-state as well as agonist-stimulated ASL pH, but only under inflammatory conditions. Furthermore, we identified two Food and Drug Administration (FDA)-approved drugs which raised ASL pH by activating SLC26A4. While we identified a role for SLC26A4 in fluid absorption, KO had no effect on cyclic adenosine monophosphate (cAMP)-stimulated fluid secretion in airway organoids. Overall, we have identified a role of TMEM16A in ASL pH homeostasis and shown that both TMEM16A and SLC26A4 could be important alternative targets for ASL pH therapy in CF, particularly for those people who do not produce any functional CFTR.

## Significance

ASL (airway surface liquid) volume and composition are strictly regulated by chloride and bicarbonate secretion via CFTR [CF (cystic fibrosis) transmembrane conductance regulator] and other transporters. In CF, the ASL is both dehydrated and acidic due to dysfunctional CFTR and ongoing proton secretion. ASL pH is, therefore, a potential factor that could be targeted for future “non-CFTR-based” CF therapies, especially for those with class I (null) mutations. In this study we were able to increase ASL pH in the absence of a functional CFTR, using two alternative transporters, TMEM16A and SLC26A4. Furthermore, we also show that two clinically-approved drugs increased ASL pH via SLC26A4, highlighting the potential role of these transporters for future ASL pH therapy in CF.

Cystic fibrosis (CF) is the most common, severe, autosomal recessive disease in the Caucasian population and is caused by mutations in the CF transmembrane conductance regulator (CFTR) gene. The predominant pathology in people with CF (pwCF) is in the lung, where the conducting airways become clogged with thick mucus, which is soon colonized by microorganisms, and this leads to cyclical, recurring inflammation. This causes structural damage to the infected tissues, bronchiectasis, and pulmonary insufficiency which ultimately ends in respiratory failure (1). Airway epithelial cells are covered by a thin liquid layer (≈8 to 10 µm in depth), the airway surface liquid (ASL) (2). The ASL is the primary innate defence against inhaled pathogens and achieves this through the active transport of mucus with trapped pathogens along the bronchial tree by mucociliary clearance (MCC) (3). Maintaining ASL homeostasis with well-hydrated mucus and clearance is vital for the lungs and keeps the airways sterile and clean (2). ASL volume and composition are strictly regulated by both sodium absorption through the epithelial sodium channel (ENaC), as well as by chloride and bicarbonate secretion via CFTR and other transporters, expressed by ciliated, goblet and club epithelial cells (4), where bicarbonate has a direct role on mucus expansion, viscosity, bacterial killing, as well as acting as a pH buffer (5). The ASL has been shown to be more acidic in cultured CF epithelia compared to non-CF (6), and the chronic recurring infections present in the airways of pwCF further acidify the ASL (7). Some of the possible players present in the apical membrane of airway cells that could induce ASL alkalinisation are the calcium-activated chloride channel (CaCC) TMEM16A (ANO1), the anion exchanger SLC26A4 (pendrin), and SLC26A9 (8), while others have a role in ASL pH acidification, such as the nongastric proton ATPase (ATP12A) and the V-type ATPase ATP6V0D2 (9).

Although there is no direct evidence that TMEM16A regulates ASL pH in humans, previous work found that the pore was flexible, leading to significant bicarbonate permeation (10), suggesting that it could potentially modulate ASL pH. TMEM16A also functionally interacts with CFTR (11) and furthermore has been proposed to work synergistically with SLC26A4 to secrete bicarbonate, a process that is up-regulated under inflammatory conditions (12). SLC26A4 is a coupled chloride and bicarbonate exchanger expressed in the apical membrane of airway cells (13), where it secretes bicarbonate after cyclic adenosine monophosphate (cAMP) stimulation (14). SLC26A4 also functionally regulates CFTR-dependent chloride secretion (15), and this functional coupling in goblet cells alkalinizes the ASL under inflammatory conditions (16, 17). In addition to TMEM16A and SLC26A4, SLC26A9 has recently been shown to regulate ASL pH in non-CF airway cells, probably by acting as a chloride/bicarbonate exchanger. Furthermore, the expression of TMEM16A, SLC26A4 and SLC26A9 are all significantly increased by Th2 cytokines, such as IL-4 and IL-13 (12, 18, 19).

In recent years, a highly effective modulator therapy (HEMT) has become available in clinics for ~90% of pwCF. However, for those who cannot benefit from this therapy because they carry severe Class I CFTR mutations, where the protein is not synthesized, or for other reasons such as variable responses, cost of the modulators, or side effects, up to 15 to 20% of all pwCF worldwide will not benefit from HEMT, and therefore, new approaches are urgently required (20). Among these, improving bicarbonate secretion to therapeutically modulate ASL pH and MCC has been suggested as an alternative therapy for CF, independent of the CF mutation (21). Therefore, the primary aim of this work was to investigate whether TMEM16A, SLC26A4, and SLC26A9 were potential targets capable of positively modulating ASL pH, in the absence of an active CFTR, under normal and inflammatory conditions. We also tested whether these alternative targets were affected by Food and Drug Administration (FDA)-approved drugs to help identify possible new therapies to restore bicarbonate secretion to CF cells as a CFTR-independent or complementary therapy for pwCF.

## Results

**Fig. 1.**
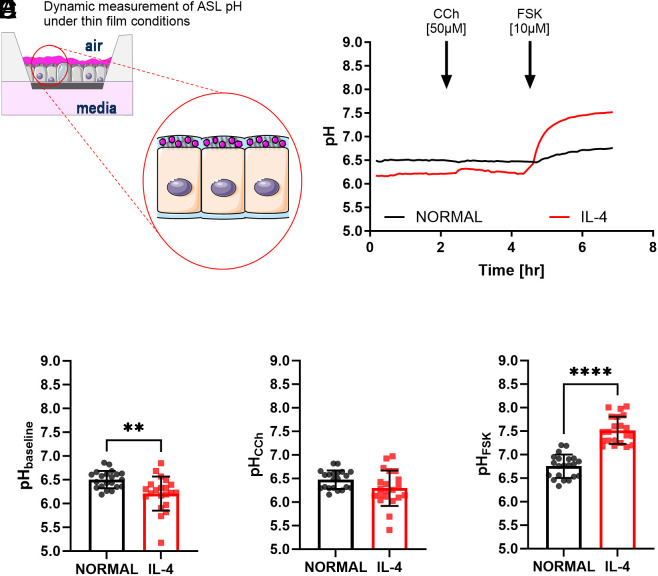
Differential effects of calcium and cAMP agonists on ASL pH in CF nasal epithelia under normal and inflammatory conditions. (*A*) Schematic overview of the fluorescent staining of the ASL with cell-impermeant pH-sensitive dye. (*B*) Summary ASL pH traces from CF epithelia under normal (black trace) or inflammatory conditions (IL-4,10 ng/mL for 48 h, red trace). Baseline ASL pH was measured for ~2 h, followed by the addition of carbachol (CCh, 50 µM), and then forskolin (FSK, 10 µM) as indicated. (*C*) Baseline ASL pH. This was calculated as the average of five time points before the addition of CCh (n = 21, three donors, unpaired *t* test). (*D*) ASL pH after acute addition of carbachol (50 µM). This was calculated as the average of five time points after the addition of CCh. (n = 21, three donors, unpaired *t* test). (*E*) ASL pH after acute addition of forskolin (10 µM). This was calculated as the average of five time points 2 h after the addition of FSK. (n = 21, three donors, unpaired *t* test). Data are presented as mean ± SD; **P < 0.01 and ****P < 0.0001.

It has already been shown that cAMP and calcium signalling can synergise to activate CFTR and other transporters and channels (22) and potentially regulate ASL pH. Here, we evaluated whether, and how, calcium and cAMP signalling could change ASL pH in the absence of CFTR under normal and inflammatory conditions. We first investigated the regulation of ASL pH in fully differentiated CF nasal epithelial cultures obtained from 3 CF donors with different class I mutations, using a pH–sensitive fluorescent dye under thin-film conditions (Fig. 1*A*), under both normal and inflammatory conditions. As shown in Fig. 1*B*, IL-4 treatment significantly reduced (*P* < 0.01) the baseline, or steady-state, ASL pH compared to epithelia under normal conditions (6.2 ± 0.4 and 6.5 ± 0.2, n = 21, respectively, Fig. 1*C*).

The muscarinic cholinergic receptor agonist, carbachol (CCh, 50 µM), increases cytosolic calcium ([Ca^2+^]_i_) in airway cells which stimulates MCC (23). Under the conditions tested here, CCh induced a significant, (*P* < 0.01), but transient, ASL alkalinisation only in IL-4 treated epithelia (ΔASL pH 0.1 ± 0.2, n = 21) as shown in the summary traces in Fig. 1*B*, which brought the ASL pH to 6.3 ± 0.4 (n = 21, Fig. 1*D*). However, post-CCh the resting ASL pH was again significantly lower (*P* < 0.01) in IL-4-treated epithelia compared to epithelia under normal conditions (6.2 ± 0.4 and 6.5 ± 0.2, n = 21, respectively). Last, the cAMP agonist, forskolin (FSK, 10 µM), caused a significant (*P* < 0.0001) and sustained alkalinisation of the ASL in both normal and inflammatory conditions (6.8 ± 0.2 and 7.5 ± 0.3, n = 21, respectively, Fig. 1 *B* and *E*). However, the FSK-induced alkalinization was greater under inflammatory (*P* < 0.0001) compared to normal conditions (ΔASL pH 1.3 ± 0.4 and 0.3 ± 0.2, n = 21, respectively). These results clearly show that IL-4-treated CF epithelia have a more acidic resting ASL pH that can be transiently increased by carbachol. Moreover, under IL-4 conditions, FSK increased ASL pH to values previously reported in the literature for non-CF ALI cultures (24), identifying an alternative (non-CFTR) bicarbonate-secretory pathway in these CF epithelia.

**Fig. 2.**
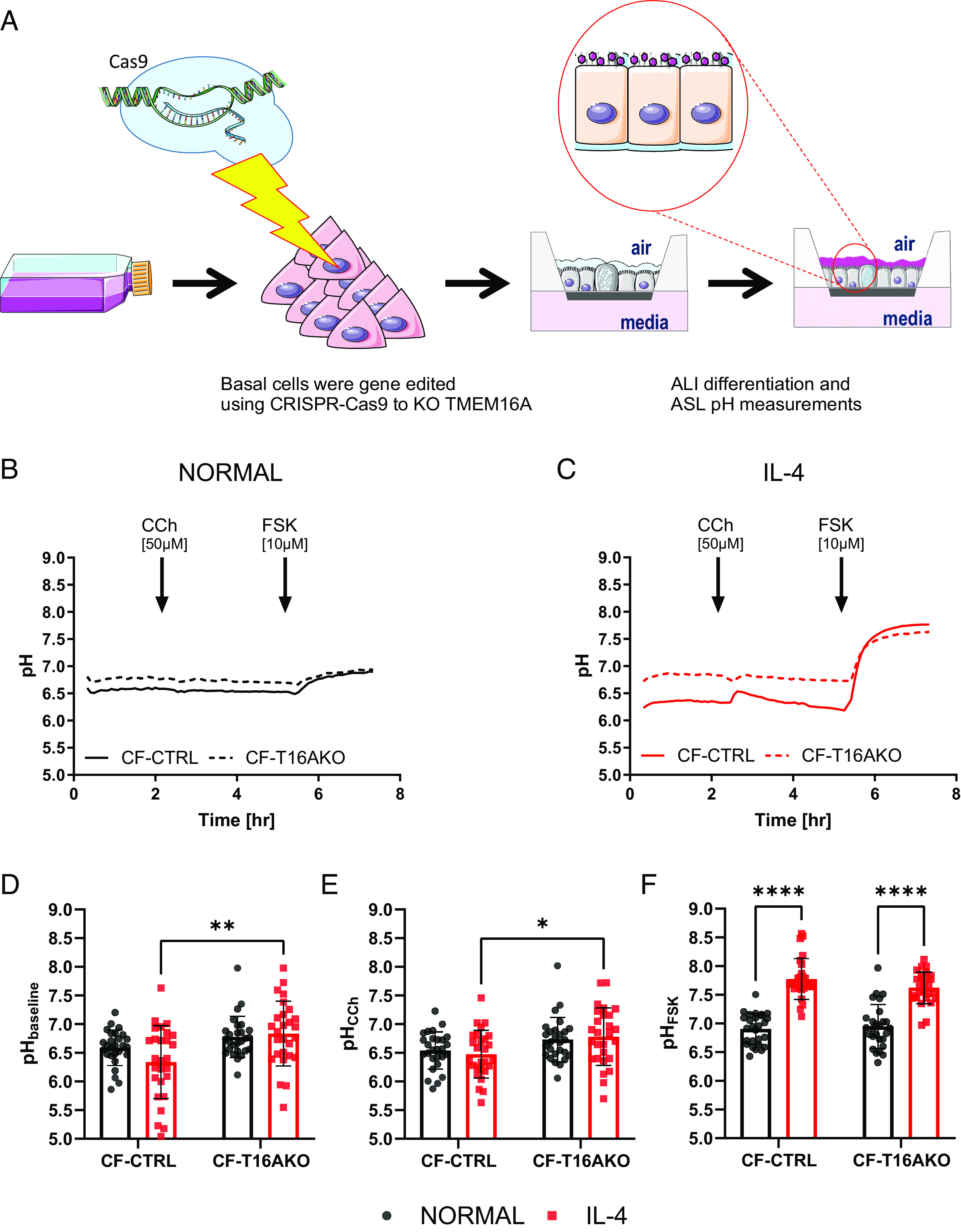
TMEM16A transiently regulates ASL pH under inflammatory conditions. (*A*) Schematic overview of the study. (*B*) Summary ASL pH traces. Solid black trace CF-CRISPRCas9 CTRL (CF) and dotted black trace CF-TMEM16AKO epithelia (CF-T16AKO) under normal conditions. (*C*) Summary ASL pH traces. Solid red trace CF-CRISPRCas9 CTRL (CF) and dotted red trace CF-TMEM16AKO (CF-T16AKO) under inflammatory conditions (IL-4, 10 ng/mL for 48 h). Baseline ASL pH was measured by adding carbachol (CCh, 50 µM), and forskolin (FSK, 10 µM) as indicated. (*D*) Baseline ASL pH. ASL pH was calculated as the average of five points before the addition of CCh. (n = 45, three donors, 2-way ANOVA with Tukey's multiple comparisons test). (*E*) CCh ASL pH. ASL pH was calculated as the average of five points after the addition of CCh. (n = 27, three donors, 2-way ANOVA with Tukey's multiple comparisons test). (*F*) FSK ASL pH. ASL pH was calculated as the average of five points 2 h after the addition of FSK. (n = 27, three donors, 2-way ANOVA with Tukey's multiple comparisons test). Data are presented as mean ± SD; *P < 0.05, **P < 0.01, and ****P < 0.0001.

TMEM16A is a CaCC that plays a role in fluid secretion in CF airways (25), as well as ASL pH regulation in the trachea of CF piglets (26). It also regulates epithelial fluid secretion and mucus clearance in human bronchial epithelial cells and in a non-CF sheep model (27). Therefore, to evaluate whether TMEM16A was directly involved in the regulation of ASL pH under normal or inflammatory conditions, TMEM16A KO and negative control nasal epithelial basal cells were generated from the 3 CF donors with the different class I mutations using CRISPR-Cas9 technology (*Materials and Methods*). The cells were then fully differentiated, and changes in ASL pH were measured as shown in the schematic overview of the protocol in Fig. 2*A*. We confirmed that efficient KO of TMEM16A had occurred by the marked reduction in short-circuit current (Isc) response to CCh in the TMEM16A KO (CF-T16AKO) epithelia compared to the CF control cultures (CF-CTRL) under both normal and inflammatory conditions (*SI Appendix*, Fig. S1).

We measured baseline ASL pH in CF-CTRL and CF-T16AKO epithelia from the 3 CF donors under normal and inflammatory conditions for over 2 h (Fig. 2 *B* and *C*). As previously found (Fig. 1*B*), IL-4 treatment reduced baseline ASL pH in CF-CTRL epithelia compared to CF-CTRL epithelia under normal conditions (6.3 ± 0.6 versus 6.6 ± 0.3, n = 27, respectively, Fig. 2*D*). In marked contrast, baseline ASL pH in CF-T16AKO epithelia treated with IL-4 was significantly (*P* < 0.01) more alkaline (6.8 ± 0.6, n = 27, Fig. 2*D*) compared to CF-CTRL epithelia treated with IL-4, suggesting that TMEM16A indirectly contributed to the acidification of ASL pH under inflammatory conditions. The subsequent addition of CCh transiently increased (*P* < 0.01) ASL pH, but only in CF-CTRL epithelia treated with IL-4 (ΔASL pH 0.1 ± 0.3, n = 27) compared to CF-CTRL epithelia under normal conditions or IL-4-treated CF-T16AKO epithelia (red trace in Fig. 2*C*). Nevertheless, the resulting ASL pH in IL-4-treated CF-CTRL epithelia was significantly lower (*P* < 0.05) compared to IL-4-treated CF-T16AKO epithelia (6.5 ± 0.4 versus 6.8 ± 0.5, n = 27, respectively, Fig. 2*E*. However, the overall change in ASL pH after 2 h was not different between the 4 conditions tested, suggesting a potential transient role for TMEM16A in ASL pH regulation, but only under IL-4 conditions.

As previously found (Fig. 1*B*), FSK induced an alkalinization of the ASL in CF-CTRL epithelia, both under normal and inflammatory conditions. Furthermore, ASL pH in IL-4-treated CF-CTRL epithelia was significantly greater (*P* < 0.0001) compared to CF-CTRL epithelia under normal conditions (7.8 ± 0.4 and 6.9 ± 0.3, n = 27, respectively, Fig. 2*F*). Also, in CF-T16AKO epithelia under inflammatory conditions, the FSK-induced alkalinization was significantly greater (*P* < 0.0001) compared to CF-T16AKO epithelia under normal conditions (7.6 ± 0.3 and 6.9 ± 0.4, n = 27 respectively, Fig. 2*F*). The fold change in ASL pH after FSK addition was maximum in IL-4-treated CF-CTRL epithelia (ΔASL pH 1.5 ± 0.9, n = 27). These results, therefore, suggest that TMEM16A has an indirect role in regulating baseline ASL pH and an active, but transient, role upon calcium stimulation, but only under IL-4 conditions. However, they exclude a role for TMEM16A in the observed FSK-induced alkalinisation under the conditions employed.

To further investigate the role of TMEM16A in the regulation of ASL pH, we tested cyclopiazonic acid (CPA, 10 µM), a mycotoxin that inhibits the endoplasmic reticulum (ER) calcium ATPase (SERCA) (28) which causes a sustained increase in [Ca^2+^]_i_. As previously found, CPA induced a sustained increase in Isc (29), and we confirmed that CPA-activated TMEM16A by measuring changes in Isc from CF control epithelial and CF-TMEM16A KO epithelia under normal and inflammatory conditions. As shown in *SI Appendix*, Fig. S2, CPA clearly activated TMEM16A, but only in IL-4-treated epithelia.

**Fig. 3.**
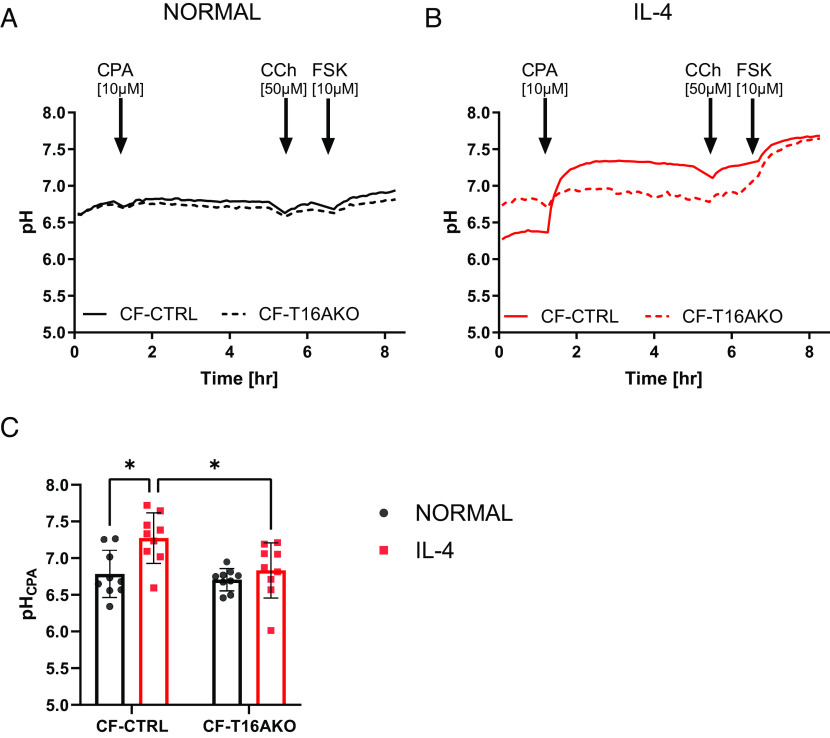
Sustained increases in cytosolic calcium raise ASL pH in CF nasal epithelia under inflammatory conditions by activating TMEM16A. (*A*) Summary ASL pH traces. Solid black trace CF-CRISPRCas9 CTRL (CF) and dotted black trace CF-TMEM16AKO epithelia (CF-T16AKO) under normal conditions. (*B*) Solid red trace CF-CRISPRCas9 CTRL (CF) and dotted red trace CF-TMEM16AKO (CF-T16AKO) under inflammatory conditions (IL-4, 10 ng/mL for 48 h). Baseline ASL pH was measured followed by the addition of CPA (10 µM) followed by carbachol (CCh, 50 µM), and then forskolin (FSK, 10 µM) as indicated. (*C*) CPA ASL pH. ASL pH was calculated as the average of five points 2 h after the addition of CPA. (n = 9, three donors, 2-way ANOVA with Tukey's multiple comparisons test). Data are presented as mean ± SD; *P < 0.05.

We then evaluated whether the acute addition of CPA also affected ASL pH and whether it was through TMEM16A. When CPA was added to CF-CTRL and CF-T16AKO epithelia under normal conditions, no change in ASL pH was observed for up to 4 h (Fig. 3*A*), consistent with Isc results. However, CPA caused a rapid, sustained, and significant (*P* < 0.0001) increase in ASL pH (ΔASL pH 0.9 ± 0.6, n = 9) in CF-CTRL epithelia under inflammatory conditions compared to CF-CTRL epithelia under normal conditions (Fig. 3*B*). Following the CPA-induced alkalinization in IL-4-treated CF-CTRL epithelia, ASL pH was significantly more alkaline (*P* < 0.05) compared to the other conditions (7.3 ± 0.3, n = 9, Fig. 3*C*). Importantly, this effect of CPA was absent in CF-T16AKO epithelia under inflammatory conditions. These results support a role of TMEM16A in regulating ASL pH, but only under inflammatory conditions. When CCh was added after CPA, no further change in ASL pH occurred under all conditions.

Finally, FSK caused a small ASL alkalinisation for both CF-CTRL and CF-T16AKO epithelia under normal conditions (0.2 ± 0.1 and 0.1 ± 0.1, n = 9). The ASL pH following the FSK-induced alkalinization reached a value of 7.7 ± 0.2 in CF-CTRL and 7.6 ± 0.3 in CF-T16AKO epithelia under inflammatory conditions. In both cases, the final ASL pH was significantly more alkaline (*P* < 0.05) compared to normal conditions. Overall, these data show that i) CPA only activates TMEM16A under inflammatory conditions and ii) TMEM16A has a direct role in ASL pH regulation when there is a sustained increase in cytosolic calcium.

In the previous ASL pH experiments, the FSK-induced ASL alkalinisation was enhanced by IL-4 pretreatment, but not affected by the KO of TMEM16A. These results suggested that SLC26A4 could be a good candidate responsible for these ASL pH responses. Therefore, SLC26A4 KO cells were made from the same CF donors with different class I mutations, and the SLC26A4 KO (CF-26A4KO) epithelia were investigated using electrophysiological and ASL pH techniques. CF-CTRL and CF-26A4KO epithelia under normal and inflammatory conditions were first tested in the Ussing chamber to evaluate whether SLC26A4 activity influenced Isc, despite it being an electroneutral anion exchanger (30). As shown in supplementary *SI Appendix*, Fig. S3, KO of SLC26A4 had no effect on Isc under any of the conditions tested.

**Fig. 4.**
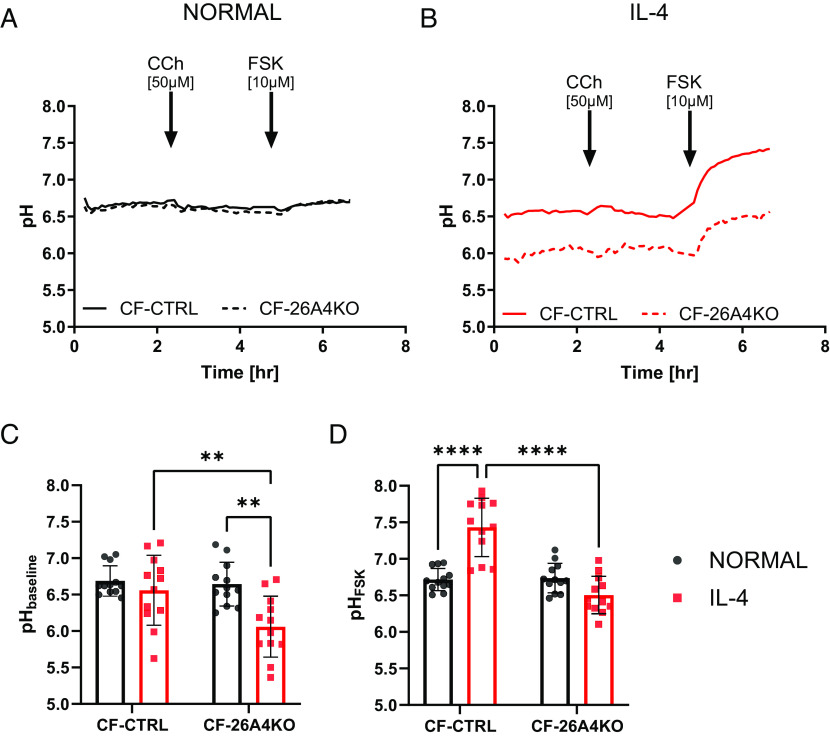
SLC26A4 regulates both baseline and forskolin-stimulated ASL pH under inflammatory conditions. (*A*) Summary ASL pH traces. *Left*, solid black trace CF-CRISPRCas9 CTRL (CF) and dotted black trace CF-SLC26A4KO epithelia (CF-26A4KO) under normal conditions. (*B*) Solid red trace CF-CRISPRCas9 CTRL (CF) and dotted red trace CF-SLC26A4KO (CF-26A4KO) under inflammatory conditions (IL-4, 10 ng/mL for 48 h). Baseline ASL pH was measured followed by the addition of carbachol (CCh, 50 µM), and then forskolin (FSK, 10 µM) as indicated. (*C*) Baseline ASL pH. ASL pH was calculated as an average of five points before the addition of CCh. (n = 12, three donors, 2-way ANOVA with Tukey’s multiple comparisons test). (*D*) FSK ASL pH. ASL pH was calculated as an average of five points after 2 h of the addition of FSK. (n = 12, three donors, 2-way ANOVA with Tukey's multiple comparisons test). Data are presented as mean ± SD; **P < 0.01 and ****P < 0.0001.

ASL pH studies showed that under normal conditions, no significant difference in resting ASL pH was found between CF-CTRL and CF-26A4KO epithelia (Fig. 4 *A* and *C*). However, under inflammatory conditions, resting ASL pH was significantly more acidic (*P* < 0.0001) in CF-26A4KO epithelial (6.1 ± 0.4, n = 12, Fig. 4*C*), compared to IL-4-treated CF-CTRL epithelia (6.6 ± 0.5, n = 12, Fig. 4*C*), and CF-CTRL epithelia under normal conditions (6.6 ± 0.3, n = 12, Fig. 4*C*). As observed before, CCh induced a small and transient increase in ASL pH in both CF-CTRL and CF-26A4KO epithelia, but only under inflammatory conditions. Immunofluorescence staining was performed to verify protein expression levels in CF CTRL and CF-26A4KO epithelia under normal and inflammatory conditions. As shown in *SI Appendix*, Fig. S5*A*, SLC26A4 was only expressed in CF-CTRL epithelia under inflammatory condition, and gene KO significantly reduced this expression consistent with the ASL pH results. Interestingly, there was strong colocalisation of SLC26A4 with MUC5AC positive cells clearly showing that the protein was expressed in goblet cells, as previously reported (12). Furthermore, this coexpression was significantly reduced (*P* < 0.0001) in CF-26A4KO compared to CF-CTRL epithelia (*SI Appendix*, Fig. S5*B*).

Under normal conditions, FSK caused a small alkalinization in both CF-CTRL and CF-26A4KO epithelia, that in CF-26A4KO epithelia significantly increased (*P* < 0.05) the ASL pH from post-CCh-treated values (6.5 ± 0.5 and 6.1 ± 0.4, n = 12, respectively). However, the changes in ASL pH were not significantly different between CF-CTRL and CF-26A4KO epithelia (ΔASL pH 0.1 ± 0.1 and 0.2 ± 0.2, n = 12, respectively, Fig. 4*D*). When FSK was added to CF-CTRL epithelia under inflammatory conditions, a significant change (*P* < 0.0001) in ASL pH occurred (ΔASL pH 0.9 ± 0.3, n = 12) compared to CF-CTRL epithelia under normal conditions (7.4 ± 0.4 and 6.7 ± 0.2, n = 12, respectively, Fig. 4*D*). Interestingly, the FSK-induced alkalinization was significantly smaller (*P* < 0.0001) (ΔASL pH 0.4 ± 0.2, n = 12, Fig. 4*D*) in CF-26A4KO epithelia compared to CF-CTRL epithelia under inflammatory conditions. Nevertheless, the ASL pH in CF-26A4KO epithelia under inflammatory conditions reached a maximum value of 6.5 ± 0.3 (n = 12, Fig. 4*D*) which was significantly more acidic (*P* < 0.001) than the ASL pH in CF-26A4KO epithelia under normal conditions, and CF-CTRL epithelia under inflammatory conditions. Interestingly, the FSK-induced alkalinisation achieved baseline ASL pH values of CF-CTRL epithelia under inflammatory conditions (Fig. 4*B*). These results suggest that SLC26A4 regulates both baseline ASL pH as well as ASL pH responses to cAMP agonists, but only under IL-4 conditions.

**Fig. 5.**
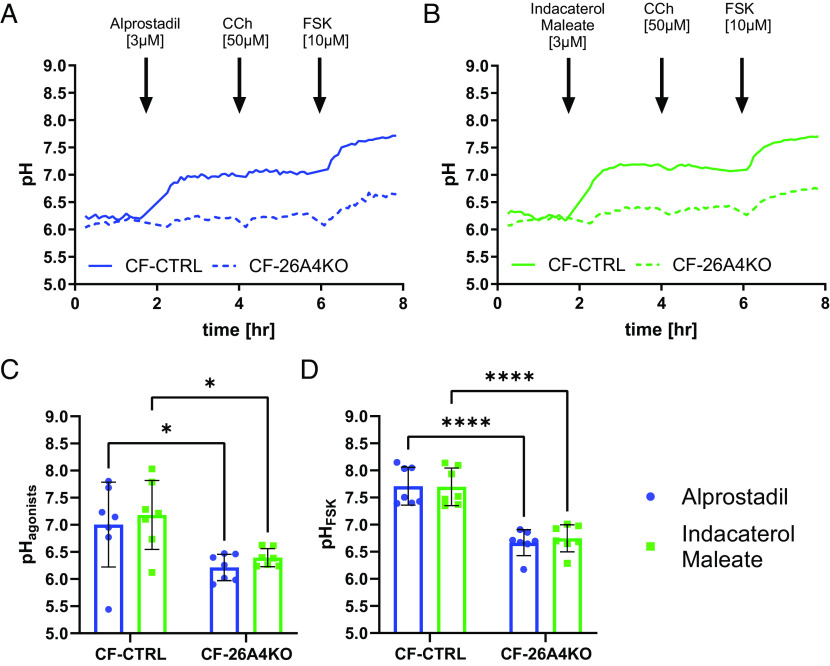
FDA-approved drugs, alprostadil and indacaterol maleate, alkalinized ASL pH under inflammatory conditions by activating SLC26A4. (*A*) Summary ASL pH traces. Solid blue trace CF-CRISPRCas9 CTRL (CF) and dotted blue trace CF-SLC26A4KO epithelia (CF-26A4KO) under normal conditions. (*B*) Summary ASL pH traces. Solid green trace CF-CRISPRCas9 CTRL (CF) and dotted green trace CF-SLC26A4KO (CF-26A4KO) under inflammatory conditions (IL-4, 10 ng/mL for 48 h). Baseline ASL pH was measured followed by the addition of alprostadil (*Left*) or indacaterol maleate (*Right*–both 3 µM). This was followed by carbachol (CCh, 50 µM) and then forskolin (FSK, 10 µM) as indicated. (*C*) ASL pH was calculated as an average of five points 2 h after the addition of alprostadil and indacaterol maleate. (n = 7, three donors, 2-way ANOVA with Tukey's multiple comparisons test). (*D*) FSK ASL pH. ASL pH was calculated as an average of five points after 2 h of the addition of FSK. (n = 7, three donors, 2-way ANOVA with Tukey’s multiple comparisons test). Data are presented as mean ± SD; *P < 0.05 and ****P < 0.0001.

SLC26A4 has previously been implicated in the β-adrenergic receptor increase in NaCl reabsorption in the kidneys (31), so we speculated that it might also be involved in the ASL pH response to FSK. Therefore, to further validate the role of SLC26A4 in ASL pH regulation under inflammatory conditions two other cAMP agonists were tested: alprostadil, a prostaglandin receptor agonist, and indacaterol maleate, a β2 adrenergic receptor agonist, which are both FDA-approved drugs. Acute addition of alprostadil and indacaterol maleate (both at 3 µM) in CF-CTRL epithelia significantly (*P* < 0.05) increased ASL pH (ΔASL pH 0.8 ± 0.8 and 1.0 ± 0.7, n = 7, respectively), a response which was completely absent in CF-26A4KO (Fig. 5*A*). After 2 h of treatment, the resulting ASL pH was significantly different (*P* < 0.05) between CF-CTRL and CF-26A4KO epithelia for both alprostadil (ASL pH 7.0 ± 0.8 and 6.2 ± 0.2, n = 7, respectively, Fig. 5*C*) and indacaterol maleate (ASL pH 7.2 ± 0.6 and 6.4 ± 0.2, n = 7, respectively, Fig. 5*C*) suggesting that these two compounds were activating SLC26A4 to increase ASL pH. The addition of CCh did not change ASL pH in any of the conditions tested (Fig. 5 *A* and *B*).

Finally, FSK (10 µM) was added to the epithelia. As shown in Fig. 5 *A* and *B*, while a FSK-induced ASL alkalinization was present in the CF-CTRL epithelia under all conditions, it was significantly smaller (*P* < 0.05) in CF-26A4KO epithelia (ΔASL pH 0.7 ± 1.0 and 0.6 ± 0.7, n = 7, respectively), as previously found. As shown in Fig. 5*D*, the ASL pH after all the stimuli, was significantly more acidic (*P* < 0.0001) in CF-26A4KO epithelia (ASL pH 6.7 ± 0.3, n = 7) compared to CF-CTRL epithelia (ASL pH 7.7 ± 0.3). These results provide strong evidence that SLC26A4 has an important role in the regulation of ASL pH under inflammatory conditions in response to physiologic cAMP agonists.

Recent work has shown that SLC26A9 contributes to ASL pH regulation in non-CF epithelia (32). SLC26A9 has been reported to be physically linked to CFTR through its STAS domain (33), and when expressed together with the CFTR mutant, F508del-CFTR, it is quickly degraded and therefore not expressed at the plasma membrane in CF cells, while in the presence of G551D-CFTR it manifests a normal trafficking phenotype (34). There is no published data regarding the expression or activity of SLC26A9 in airway cells derived from people with class I CF mutations. Therefore, we investigated whether SLC26A9 contributed to Isc and ASL pH in CF cells with class I mutations under normal and inflammatory conditions, using CF-CTRL and SLC26A9 KO (CF-26A9KO) epithelia.

As shown in *SI Appendix*, Fig. S4 *A* and *B*, no SLC26A9-dependent activity was identified in Isc experiments. The lack of any functional SLC26A9 was also confirmed in ASL pH experiments, as shown in *SI Appendix*, Fig. S4 *C*–*F*. Here, resting ASL pH was 6.44 ± 0.30 (n = 9, *SI Appendix*, Fig. S4*E*) in CF-26A9KO epithelia under normal conditions and 6.37 ± 0.74 (n = 9, *SI Appendix*, Fig. S4*E*) under inflammatory conditions, suggesting no basal activity of SLC26A9 in these cells. CCh caused no response in both CF-CTRL and CF-26A9KO epithelia, while a transient ASL pH alkalinization was found under inflammatory conditions. Finally, FSK-induced an alkalinisation which brought the ASL pH of CF-CTRL and CF-26A9KO epithelia under the inflammatory conditions to 7.70 ± 0.14 and 7.63 ± 0.09, n = 9, respectively (*SI Appendix*, Fig. S4*F*), significantly more alkaline (*P* < 0.01) compared to CF-CTRL and CF-26A9KO epithelia under normal conditions (6.75 ± 0.37 and 6.79 ± 0.25, n = 9, respectively, *SI Appendix*, Fig. S4*F*). The absence of any difference between the two types of epithelia suggests that there was no functional SLC26A9 protein in these epithelia, presumably due to the lack of any CFTR.

## Discussion

Restoring ASL pH homeostasis is a promising therapy for CF as an acid pH has been shown to increase ENaC-mediated fluid absorption and reduce mucin secretion and expansion as well as microbial killing (13). CF airways have a lower ASL pH due to the lack of CFTR-dependent bicarbonate secretion and ongoing proton secretion (35). Therefore, correcting ASL pH should improve mucus hydration, expansion, and MCC, thereby reducing microbial infections (36). Different transport proteins, in addition to CFTR, have been proposed to play a fundamental role in the regulation of ASL pH: SLC26A9, SLC26A4 (pendrin), nongastric proton pumps, ENaC, and TMEM16A to mention a few (8). Hence, correcting ASL pH could play a crucial role in improving MCC and be a reasonable alternative therapy for pwCF (20, 37).

Under normal conditions, CF epithelia with Class I mutations did not respond to CCh, and ASL pH was slightly more alkaline after the addition of FSK. However, under inflammatory conditions, resting ASL pH was significantly lower compared to normal conditions suggesting an increased secretion of protons, perhaps by an active ATP12A or sodium/hydrogen exchanger (NHE) (12, 16). The ASL pH was then further increased by FSK (Fig. 1*B*). Given that no CFTR activity was measured in Isc studies it is unlikely that the forskolin-induced alkalinization was due to CFTR, and was therefore due to another transporter of which SLC26A4 (pendrin), a chloride/bicarbonate exchanger was the main candidate, as it is overexpressed in the presence of IL-4 (19) and it is activated in response to a cAMP agonist (14).

TMEM16A has been proposed to regulate fluid secretion in the airways (25), but whether it can modulate ASL pH is still hotly debated (38). In our ASL pH studies, baseline pH was surprisingly found to be more alkaline in CF-T16AKO epithelia under inflammatory conditions contrary to what was measured in CF-CTRL epithelia (Fig. 2*D*), suggesting that TMEM16A had an indirect role in the regulation of resting ASL pH, perhaps via regulating other CaCC, or members of the CLCA family (39). The calcium agonist carbachol induced a transient, but TMEM16A-dependent, increase in ASL pH, but only under inflammatory conditions. However, Isc studies found that CCh was a weak stimulator of TMEM16A (*SI Appendix*, Fig. S1*B*). The weak effect of CCh on ASL pH/Isc is consistent with a small calcium response. Consistent with this hypothesis, it was found that the SERCA pump inhibitor CPA (28), which causes a sustained increase in cytosolic calcium levels (40), only increased ASL pH in CF epithelia under inflammatory conditions, clearly showing that TMEM16A was capable of regulating ASL pH, but only when it is up-regulated (Fig. 3*C*). This demonstrates that TMEM16A is directly involved in ASL pH regulation in human airway cells. In support of this finding, CPA also increased Isc in CF-CTRL epithelia under inflammatory conditions, which was absent in CF-T16AKO epithelia (*SI Appendix*, Fig. S2*B*), clearly proving that TMEM16A was involved in the CPA response. Future work should identify molecules that could act as direct TMEM16A openers to mimic the CPA response identified under inflammatory conditions.

Under normal conditions, the absence of SLC26A4 did not affect baseline ASL pH. However, under inflammatory conditions, KO of SLC26A4 markedly acidified baseline ASL pH (Fig. 4*C*), suggesting that it was active under resting conditions. It has been described previously that SLC26A4 is regulated by both intracellular and extracellular pH (41), Therefore, since the baseline ASL pH was more acidic in CF epithelia under inflammatory conditions (Fig. 1*B*), this may have caused the increased SLC26A4 activity. Furthermore, SLC26A4 was also found to underlie most of the FSK-induced alkalinisation under inflammatory conditions (Fig. 4*D*), one of the most unexpected results obtained in these CF epithelia with class I mutations. This clearly illustrates the important role that SLC26A4 has in the regulation of ASL pH, consistent with findings from other groups (13, 17). Simonin et al. showed that SLC26A4 activity was impaired in the presence of the F508del mutation (13) since the two proteins functionally couple together to increase ion secretion (15). Our work demonstrates that SLC26A4 was active in the absence of any functional CFTR (no CFTR activity based on Isc experiments in *SI Appendix*, Figs. S3 and S4). This result is particularly interesting as it has been shown that other SLC26A transporters (SLC26A3, A6) require CFTR expression at the plasma membrane to function (42, 43), which agrees with our results with SLC26A9, whose activity was not detected in these epithelia with class I mutations. The lack of SLC26A9 activity, in the complete absence of CFTR, in fully differentiated airway epithelial cells has important implications for using this protein as a potential target for non-CFTR therapies.

However, SLC26A4 was not the only transporter responsible for the FSK-induced alkalinisation, as evidenced by the residual alkalinisation measured in the CF-26A4KO epithelia. This suggests that other cAMP-activated channels or transporters were active, or other pathways involved; for example, paracellular transport has been suggested to be compromised in airway diseases (44), or, as recently suggested, alkalinisation could be due to the β-adrenergic-induced inhibition of the proton pump ATP12A (16), which is overexpressed by IL-4 treatment, and actively acidified ASL pH (45). In addition, cAMP may also lead to the removal (internalisation) of apical NHE to promote ASL alkalinisation, as previously demonstrated in CF gastrointestinal cells (46). Future work will be required to investigate these possibilities. Since SLC26A4 has been implicated in the β-adrenergic receptor response in the kidneys (31), we evaluated whether this was also the case in the airways and whether the response involved SLC26A4 or ATP12A (16). We tested a prostaglandin receptor agonist, alprostadil, and the β_2_ receptor agonist, indacaterol maleate, under inflammatory conditions. Both FDA-approved drugs significantly increased ASL pH in CF control epithelia (Fig. 5*C*), but this effect was absent in SLC26A4 KO epithelia, suggesting a direct involvement of this anion exchanger in the response. Interestingly, both drugs stimulated fluid secretion in our recent high throughput screening of an FDA drug library, using CF nasal AO (47). This further highlights the important role of bicarbonate secretion in CF.

While our results suggest that SLC26A4-mediated HCO_3_^–^ secretion would be beneficial for CF airways, other studies have implicated this transporter in fluid absorption, as shown in airway cells from patients with Pendred Syndrome, where the SLC26A4 gene is mutated, but CFTR is still active. In these epithelia, ASL volume was greater compared to healthy controls (48). More recent work from Haggie et al. (49) and Guidone et al. (16) found that exposure to pharmacological inhibitors of SLC26A4 increased ASL height, or decreased mucus viscosity, respectively, in primary cultures of airway cells (non-CF and CF). Based on these results, the authors proposed that inhibiting SLC26A4 would be a good strategy for improving fluid homeostasis and MCC in CF. However, the two papers produced conflicting results with respect to ASL pH. While the Guidone paper showed that pharmacologically inhibiting SLC26A4 reduced ASL pH, as expected for a Cl^−^/HCO_3_^−^ exchanger, the Haggie study showed no such effect. In order to help clarify the role of SLC26A4 in ASL fluid homeostasis, we generated nasal organoids from the same CF donors used for the ASL pH and ion transport studies and evaluated the effect of SLC26A4 KO on fluid homeostasis. As described earlier, we have recently shown that these AO swell over time in response to cAMP agonists due to a CFTR and TMEM16A-independent intraluminal accumulation of fluid (47). We reasoned that if SLC26A4 was involved in fluid absorption, then KO of this transporter would be predicted to enhance the rate and/or the extent of AO swelling in response to cAMP, based on the findings of Haggie and Guidone. However, *SI Appendix*, Fig. S6 *A*–*C* clearly shows that KO of SLC26A4 had no significant effect on the rate or magnitude of cAMP-stimulated organoid swelling in the presence of IL-4. When taken together with our ASL pH results (Figs. 4 and 5), these data provide strong evidence that upon cAMP stimulation, SLC26A4 activity does limit fluid secretion, but promotes HCO_3_^−^ secretion in airway cells which lack CFTR. However, careful examination of individual organoids under the different conditions, showed there was a significant decrease in mean organoid area (size) in the IL-4 treated organoids which was abrogated by KO of SLC26A4 (*SI Appendix*, Fig. S6*D*). This latter result suggests that under steady-state conditions (i.e., in the absence of cAMP stimulation) SLC26A4 was involved in fluid absorption, consistent with the Haggie and Guidone studies (16, 49). We therefore suggest, that, in the presence of cAMP stimulation SLC26A4 switches from an absorptive to a secretory role. This cAMP-dependent switch in function has previously been described in small intestinal cells, where the related anion exchanger, SLC26A3 works together with NHE3, to accomplish net NaCl absorption, but this functional coupling is lost by cAMP stimulation, and SLC26A3 then works with CFTR to promote NaHCO_3_ and fluid secretion (46).

While our results are clearly at odds with those studies that suggest SLC26A4 should be inhibited to improve airway hydration (16, 49), it is possible that the complete absence of CFTR may affect the functional “activity” of SLC26A4 itself, as we have recently shown for the related family member SLC26A9 in the airways (32). The lack of CFTR could also impact the functional “coupling” between SLC26A4 and other proteins that it may interact with to accomplish net fluid absorption (such as ENaC or NHE). This is relevant because both the Haggie and Guidone studies used CF airway cells derived from people homozygous for F508del-CFTR, and as shown by Simonin et al. (13), F508del-CFTR reduced SLC26A4 activity. Therefore, future work could be performed in bronchial epithelial cells, where CFTR is not expressed. It will also be important to assess whether SLC26A4 regulates fluid absorption under different physiological conditions and whether other proteins interact with SLC26A4 to enable it to carry out its anion exchange activity. The approach we have described here, using gene-edited nasal basal cells, has provided interesting insights about the physiology of airway epithelia ASL pH homeostasis, in the complete absence of CFTR, and should facilitate these future studies complementing pharmacological inhibitor work which will be important for developing alternative therapies for these Class I mutations.

In summary, our work shows that SLC26A4 was active in CF epithelia with Class I mutations where it directly regulated steady-state as well as FSK-induced alkalinisation under inflammatory conditions. In addition, our organoid swelling results provided no evidence that this transporter had a negative effect on cAMP-dependent fluid secretion. Thus, under physiological conditions in vivo, where endogenous cAMP agonists such as ATP, adenosine, vasoactive intestinal peptide, and adrenaline will be active in the airways, inhibiting SLC26A4 would not be beneficial as it would reduce ASL pH, and potentially limit maximal fluid secretion in pwCF with Class I mutations. Overall, these results show that SLC26A4 has an important role in regulating ASL homeostasis in the absence of an active CFTR. Moreover, our work also demonstrated that TMEM16A has both a direct and indirect role in the ASL pH regulation under inflammatory conditions and should still be considered a good alternative target for CF ASL pH therapy, especially since TMEM16A expression has recently been found not to correlate with mucin overproduction under inflamed conditions (50). More work must be done to evaluate the potential interaction between SLC26A4 and TMEM16A in CF cells with class I mutations, as both could act synergistically to improve ASL pH under inflammatory conditions.

## Materials and Methods

### Chemicals.

Amiloride (A7410) and carbamylcholine chloride (C4382) were purchased from Sigma-Aldrich. Forskolin (1099) and CPA (1235) were purchased from Bio-Techne. Alprostadil (S1508) and Indacaterol Maleate (S3083) were purchased from Stratech Scientific Ltd.

### Solutions.

For the physiological assays, we used an HCO_3_^−^ KRB solution containing in mM: 25 NaHCO_3_, 115 NaCl, 5 KCl, 1 CaCl_2_, 1 MgCl_2_, and 5 D-glucose, pH 7.4, at 37 °C. ASL pH standard calibration solutions were modified Ringer solution containing, in mM, 86 NaCl, 5 KCl, 1.2 CaCl_2_, 1.2 MgCl_2_, and either 50 MES (pH 5.5), 50 HEPES (pH 7.0), or 50 Tris (pH 8.0) at 37 °C.

### Cell Culture.

Primary CF basal epithelial cells derived from nasal brushings were obtained from 3 donors with the following class I CFTR mutations: W1282X/1717-1G>A; R553X/R553X; G542X/Dele2.3 (21 kb). Nasal brushings were collected from donors after obtaining their informed consent. The full study protocol was approved by the biobanking-dedicated ethical review board (TcBIO) of the University Medical Center Utrecht under protocol TcBIO ID: 19/764 (see *SI Appendix* for more details). Nasal epithelial cells were expanded and differentiated as previously described (47). See *SI Appendix* for more details.

### Knock-Out of TMEM16A, SLC26A4, and SLC26A9 Using CRISPR-Cas9.

TMEM16A, SLC26A4, and SLC26A9 KO primary epithelial cells from the 3 CF donors were created using the CRISPR-Cas9 method, as previously published (47). The gene editing efficiency is presented in *SI Appendix*, Fig. S7 for SLC26A4 and SLC26A9 KO, while TMEM16A KO data are described in the study by Rodenburg et al. (47). See *SI Appendix* for more details.

### ASL pH Measurements.

ASL pH was measured using a temperature and CO_2_-controlled plate reader (TECAN SPARK 10M), as previously described (51). See *SI Appendix* for more details.

### Short-Circuit Current Measurements.

Epithelial cultures were mounted into the EasyMount Ussing Chamber System (VCC MC8, Physiologic Instrument). The transepithelial short-circuit current (Isc) was recorded as previously described (52). See *SI Appendix* for more details.

### Immunofluorescence Assay.

Epithelia cultures were stained to evaluate SLC26A4, MUC5AC, and actin protein expression and localization under the condition tested. See *SI Appendix* for more details.

### Generation of AO and Organoid Swelling Assay.

Differentiated ALI HNEC epithelia were used to generate AO, and AO swelling was performed as previously described (47, 53). See *SI Appendix* for more details.

### Statistical Analysis.

Statistical analysis was performed using GraphPad Prism 9 software. Data are presented as mean ± SD. Multiple-group and two-group comparisons were performed using appropriate statistical tests for specific datasets (see details in individual figure legends).

## Supplementary Material

Appendix 01 (PDF)

## Data Availability

All data generated during this research are openly available at https://doi.org/10.25405/data.ncl.24085257.v1 (54). All other data are included in the manuscript and/or *SI Appendix*.

## References

[1] J. S. Elborn, Cystic fibrosis. Lancet **388**, 2519–2531 (2016). 27140670 10.1016/S0140-6736(16)00576-6

[2] M. A. Mall, Role of cilia, mucus, and airway surface liquid in mucociliary dysfunction: Lessons from mouse models. J. Aerosol Med. Pulm. Drug Deliv. **21**, 13–24 (2008). 18518828 10.1089/jamp.2007.0659

[3] Y. Xie , Mucociliary transport in healthy and cystic fibrosis pig airways. Ann. Am. Thorac. Soc. **15**, S171–S176 (2018). 30431346 10.1513/AnnalsATS.201805-308AW PMC6322029

[4] Y. Wang, J. A. Wrennall, Z. Cai, H. Li, D. N. Sheppard, Understanding how cystic fibrosis mutations disrupt CFTR function: From single molecules to animal models. Int. J. Biochem. Cell Biol. **52**, 47–57 (2014). 24727426 10.1016/j.biocel.2014.04.001

[5] K. Kunzelmann, R. Schreiber, H. B. Hadorn, Bicarbonate in cystic fibrosis. J. Cyst. Fibros. **16**, 653–662 (2017). 28732801 10.1016/j.jcf.2017.06.005

[6] R. D. Coakley , Abnormal surface liquid pH regulation by cultured cystic fibrosis bronchial epithelium. Proc. Natl. Acad. Sci. U.S.A. **100**, 16083–16088 (2003). 14668433 10.1073/pnas.2634339100 PMC307696

[7] T. Rehman, M. J. Welsh, Inflammation as a regulator of the airway surface liquid pH in cystic fibrosis. Cells **12**, 1104 (2023). 37190013 10.3390/cells12081104 PMC10137218

[8] M. Zajac, E. Dreano, A. Edwards, G. Planelles, I. Sermet-Gaudelus, Airway surface liquid pH regulation in airway epithelium current understandings and gaps in knowledge. Int. J. Mol. Sci. **22**, 3384 (2021). 33806154 10.3390/ijms22073384 PMC8037888

[9] X. Li , V-Type ATPase mediates airway surface liquid acidification in pig small airway epithelial cells. Am. J. Respir. Cell Mol. Biol. **65**, 146–156 (2021). 33789071 10.1165/rcmb.2020-0349OC PMC8399571

[10] I. Jun , Pore dilatation increases the bicarbonate permeability of CFTR, ANO1 and glycine receptor anion channels. J. Physiol. **594**, 2929–2955 (2016). 26663196 10.1113/JP271311 PMC4887682

[11] J. Lerias , Compartmentalized crosstalk of CFTR and TMEM16A (ANO1) through EPAC1 and ADCY1. Cell. Signal. **44**, 10–19 (2018). 29331508 10.1016/j.cellsig.2018.01.008

[12] G. Gorrieri , Goblet cell hyperplasia requires high bicarbonate transport to support mucin release. Sci. Rep. **6**, 36016 (2016). 27786259 10.1038/srep36016 PMC5081536

[13] J. Simonin , Airway surface liquid acidification initiates host defense abnormalities in cystic fibrosis. Sci. Rep. **9**, 6516 (2019). 31019198 10.1038/s41598-019-42751-4 PMC6482305

[14] J. P. Garnett , Novel role for pendrin in orchestrating bicarbonate secretion in cystic fibrosis transmembrane conductance regulator (CFTR)-expressing airway serous cells. J. Biol. Chem. **286**, 41069–41082 (2011). 21914796 10.1074/jbc.M111.266734 PMC3220502

[15] J. Bajko, M. Duguid, S. Altmann, G. D. Hurlbut, J. S. Kaczmarek, Pendrin stimulates a chloride absorption pathway to increase CFTR-mediated chloride secretion from Cystic Fibrosis airway epithelia. FASEB Bioadv. **2**, 526–537 (2020). 32923987 10.1096/fba.2020-00012 PMC7475303

[16] D. Guidone , Airway surface hyperviscosity and defective mucociliary transport by IL-17/TNF-alpha are corrected by beta-adrenergic stimulus. JCI Insight **7**, e164944 (2022), 10.1172/jci.insight.164944. 36219481 PMC9746827

[17] T. Rehman , TNFalpha and IL-17 alkalinize airway surface liquid through CFTR and pendrin. Am. J. Physiol. Cell Physiol. **319**, C331–C344 (2020). 32432926 10.1152/ajpcell.00112.2020 PMC7500220

[18] J. Ousingsawat, R. Centeio, R. Schreiber, K. Kunzelmann, Expression of SLC26A9 in airways and its potential role in Asthma. Int. J. Mol. Sci. **23**, 2998 (2022). 35328418 10.3390/ijms23062998 PMC8950296

[19] S. Vanoni, G. Scantamburlo, S. Dossena, M. Paulmichl, C. Nofziger, Interleukin-mediated pendrin transcriptional regulation in airway and esophageal epithelia. Int. J. Mol. Sci. **20**, 731 (2019). 30744098 10.3390/ijms20030731 PMC6386862

[20] L. Allen , Future therapies for cystic fibrosis. Nat. Commun. **14**, 693 (2023). 36755044 10.1038/s41467-023-36244-2 PMC9907205

[21] X. X. Tang , Acidic pH increases airway surface liquid viscosity in cystic fibrosis. J. Clin. Invest. **126**, 879–891 (2016). 26808501 10.1172/JCI83922 PMC4767348

[22] Z. Bozoky , Synergy of cAMP and calcium signaling pathways in CFTR regulation. Proc. Natl. Acad. Sci. U.S.A. **114**, E2086–E2095 (2017). 28242698 10.1073/pnas.1613546114 PMC5358358

[23] N. S. Joo, M. E. Krouse, J. Y. Choi, H. J. Cho, J. J. Wine, Inhibition of airway surface fluid absorption by cholinergic stimulation. Sci. Rep. **6**, 20735 (2016). 26846701 10.1038/srep20735 PMC4742893

[24] D. Kim , Large pH oscillations promote host defense against human airways infection. J. Exp. Med. **218**, e20201831 (2021). 33533914 10.1084/jem.20201831 PMC7845918

[25] H. Danahay, M. Gosling, TMEM16A: An alternative approach to restoring airway anion secretion in cystic fibrosis? Int. J. Mol. Sci. **21**, 2386 (2020). 32235608 10.3390/ijms21072386 PMC7177896

[26] R. Benedetto , Transport properties in CFTR-/- knockout piglets suggest normal airway surface liquid pH and enhanced amiloride-sensitive Na(+) absorption. Pflugers Arch. **472**, 1507–1519 (2020). 32712714 10.1007/s00424-020-02440-y PMC7476968

[27] H. L. Danahay , TMEM16A potentiation: A novel therapeutic approach for the treatment of cystic fibrosis. Am. J. Respir. Crit. Care Med. **201**, 946–954 (2020). 31898911 10.1164/rccm.201908-1641OC PMC7159426

[28] N. W. Seidler, I. Jona, M. Vegh, A. Martonosi, Cyclopiazonic acid is a specific inhibitor of the Ca2+-ATPase of sarcoplasmic reticulum J. Biol. Chem. **264**, 17816–17823 (1989). 2530215

[29] C. Schwarzer , Thapsigargin blocks Pseudomonas aeruginosa homoserine lactone-induced apoptosis in airway epithelia. Am. J. Physiol. Cell Physiol. **306**, C844–C855 (2014). 24598360 10.1152/ajpcell.00002.2014 PMC4010806

[30] N. Shcheynikov , The Slc26a4 transporter functions as an electroneutral Cl-/I-/HCO3- exchanger: Role of Slc26a4 and Slc26a6 in I- and HCO3- secretion and in regulation of CFTR in the parotid duct. J. Physiol. **586**, 3813–3824 (2008). 18565999 10.1113/jphysiol.2008.154468 PMC2538934

[31] A. Azroyan , Regulation of pendrin by cAMP: Possible involvement in beta-adrenergic-dependent NaCl retention. Am. J. Physiol. Renal Physiol. **302**, F1180–F1187 (2012). 22262479 10.1152/ajprenal.00403.2011

[32] S. Jo , The SLC26A9 inhibitor S9–A13 provides no evidence for a role of SLC26A9 in airway chloride secretion but suggests a contribution to regulation of ASL pH and gastric proton secretion. FASEB J. **36**, e22534 (2022). 36183361 10.1096/fj.202200313RR

[33] M. Avella, C. Loriol, K. Boulukos, F. Borgese, J. Ehrenfeld, SLC26A9 stimulates CFTR expression and function in human bronchial cell lines. J. Cell Physiol. **226**, 212–223 (2011). 20658517 10.1002/jcp.22328

[34] Y. Sato, D. Y. Thomas, J. W. Hanrahan, The anion transporter SLC26A9 localizes to tight junctions and is degraded by the proteasome when co-expressed with F508del-CFTR. J. Biol. Chem. **294**, 18269–18284 (2019). 31645438 10.1074/jbc.RA119.010192 PMC6885613

[35] L. Delpiano , Esomeprazole increases airway surface liquid pH in primary cystic fibrosis epithelial cells. Front. Pharmacol. **9**, 1462 (2018). 30618754 10.3389/fphar.2018.01462 PMC6297391

[36] I. J. Haq, M. A. Gray, J. P. Garnett, C. Ward, M. Brodlie, Airway surface liquid homeostasis in cystic fibrosis: Pathophysiology and therapeutic targets. Thorax **71**, 284–287 (2016). 26719229 10.1136/thoraxjnl-2015-207588

[37] A. Schultz , Airway surface liquid pH is not acidic in children with cystic fibrosis. Nat. Commun. **8**, 1409 (2017). 29123085 10.1038/s41467-017-00532-5 PMC5680186

[38] K. Kunzelmann , TMEM16A in cystic fibrosis: Activating or inhibiting? Front. Pharmacol. **10**, 3 (2019). 30761000 10.3389/fphar.2019.00003 PMC6362895

[39] A. Sharma, G. Ramena, Y. Yin, L. Premkumar, R. C. Elble, CLCA2 is a positive regulator of store-operated calcium entry and TMEM16A. PLoS One **13**, e0196512 (2018). 29758025 10.1371/journal.pone.0196512 PMC5951673

[40] R. Centeio , Pharmacological inhibition and activation of the Ca(2+) activated Cl(-) channel TMEM16A. Int. J. Mol. Sci. **21**, 2557 (2020). 32272686 10.3390/ijms21072557 PMC7177308

[41] A. Azroyan , Regulation of pendrin by pH: Dependence on glycosylation. Biochem. J. **434**, 61–72 (2011). 21073444 10.1042/BJ20101411

[42] Y. Wang , Slc26a6 regulates CFTR activity in vivo to determine pancreatic duct HCO3- secretion: Relevance to cystic fibrosis. EMBO J. **25**, 5049–5057 (2006). 17053783 10.1038/sj.emboj.7601387 PMC1630422

[43] P. Hegyi , SLC26 transporters and the inhibitory control of pancreatic ductal bicarbonate secretion. Novartis Found. Symp. **273**, 164–173; discussion 173–166, 261–164 (2006). 17120767

[44] K. E. Scheckenbach , Prostaglandin E(2)regulation of cystic fibrosis transmembrane conductance regulator activity and airway surface liquid volume requires gap junctional communication. Am. J. Respir. Cell Mol. Biol. **44**, 74–82 (2011). 20167933 10.1165/rcmb.2009-0361OC PMC3028258

[45] P. Scudieri , Increased expression of ATP12A proton pump in cystic fibrosis airways. JCI Insight **3**, e123616 (2018). 30333310 10.1172/jci.insight.123616 PMC6237449

[46] X. Tan, A. Kini, D. Romermann, U. Seidler, The NHE3 inhibitor tenapanor prevents intestinal obstructions in CFTR-deleted mice. Int. J. Mol. Sci. **23**, 9993 (2022). 36077390 10.3390/ijms23179993 PMC9456459

[47] L. W. Rodenburg , Drug repurposing for cystic fibrosis: Identification of drugs that induce CFTR-independent fluid secretion in nasal organoids. Intern. J. Mol. Sci. **23**, 12657 (2022). 10.3390/ijms232012657 PMC9603984 36293514

[48] H. J. Lee , Thick airway surface liquid volume and weak mucin expression in pendrin-deficient human airway epithelia. Physiol. Rep. **3**, e12480 (2015). 26243215 10.14814/phy2.12480 PMC4562566

[49] P. M. Haggie , Inhibitors of pendrin anion exchange identified in a small molecule screen increase airway surface liquid volume in cystic fibrosis. FASEB J. **30**, 2187–2197 (2016). 26932931 10.1096/fj.201600223R PMC4871793

[50] F. B. Simoes , TMEM16A chloride channel does not drive mucus production. Life Sci. Alliance **2**, e201900462 (2019). 31732694 10.26508/lsa.201900462 PMC6859295

[51] V. Saint-Criq , Real-time, semi-automated fluorescent measurement of the airway surface liquid pH of primary human airway epithelial cells. J. Vis. Exp. **148**, 1–9 (2019). 10.3791/59815 PMC6748865 31259916

[52] V. Saint-Criq , Choice of differentiation media significantly impacts cell lineage and response to CFTR modulators in fully differentiated primary cultures of cystic fibrosis human airway epithelial cells. Cells **9**, 2137 (2020). 32967385 10.3390/cells9092137 PMC7565948

[53] G. D. Amatngalim , Measuring cystic fibrosis drug responses in organoids derived from 2D differentiated nasal epithelia. Life Sci. Alliance **5**, e202101320 (2022). 35922154 10.26508/lsa.202101320 PMC9351388

[54] M.A. Gray, L. Delpiano, Data for 2023 PNAS publication on airway surface liquid (ASL) pH in cystic fibrosis (CF) human airway cells. data.ncl.ac.uk. 10.25405/data.ncl24085257.v1. Deposited 10 October 2023.

