# Supplementary material for: Dynamic regulation of airway surface liquid pH by TMEM16A and SLC26A4 in cystic fibrosis nasal epithelia with rare mutations
Source: Proc Natl Acad Sci U S A. 2023 Nov 15;120(47):e2307551120. doi: 10.1073/pnas.2307551120 (PMC10666107; doi:10.1073/pnas.2307551120)
Supplement: Supplementary file 1 — Appendix 01 (PDF) [file pnas.2307551120.sapp.pdf]

PNAS

Supporting Information for

Dynamic regulation of airway surface liquid pH by TMEM16A and SLC26A4 in cystic fibrosis nasal epithelia with rare mutations.

Livia Delpiano[1], Lisa W. Rodenburg[3,4], Matthew Burke[1], Glyn Nelson[2], Gimano D. Amatngalim[3,4], Jeffrey M. Beekman[3,4,5], Michael A. Gray[1].

Corresponding author: Michael A Gray.

Email: m.a.gray@newcastle.ac.uk

This PDF file includes:

Supporting text

Figures S1 to S7

Extended Materials and Methods

Supporting Information Text

Functional validation of CRISPR-Cas9 KO of TMEM16A, SLC26A4 and SLC26A9 using short circuit (Isc) and ASL pH measurements.

Effect of carbachol in CF-CTRL and CF-T16AKO nasal epithelia.

In the presence of amiloride (10µM), CCh induced a small increase in Isc, which was greater in CF-CTRL epithelia compared to CF-T16AKO epithelia (ΔIsc 2.3±1.7 and 0.6±0.6 µAmp*cm^{-2}, n=10, respectively, Figure S1B). Under inflammatory conditions, CCh caused a markedly increased Isc response in CF-CTRL epithelia, which was completely abrogated in CF-T16AKO epithelia (p<0.0001, ΔIsc 0.3±0.3 µAmp*cm^{-2}, n=9, Figure S1B). There was no difference in the CCh-induced Isc between CF-T16AKO epithelia under normal or inflammatory conditions suggesting that TMEM16A underlies most of the response to CCh.

Effect of CPA in CF-CTRL and CF-T16AKO nasal epithelia.

CPA (10µM) was tested basolaterally on CF-CTRL and CF-T16AKO epithelia, in the presence of amiloride (10µM). Under normal conditions, for both CF-CTRL and CF-T16AKO epithelia, CPA did not change Isc, as shown in the representative traces in Figure S2A left panel. Note that a slow drift in Isc was present for both CF-CTRL and CF-T16AKO epithelia (ΔIsc 1.7±1.0 and 2.4±1.9 µAmp*cm^{-2}, n=6, respectively, Figure S2B). However, it was not affected by the basolateral addition of CPA, suggesting that it was not due to the activity of a CaCC. In contrast, when CPA was added to CF-CTRL epithelia under inflammatory conditions, it caused a significant increase in Isc (ΔIsc 5.2±2.5 µAmp*cm^{-2}, n=6, p<0.01, Figure S2B), compared to CF-CTRL epithelia under normal conditions, as shown in the representative red trace in Figure S2A right panel, and in the summary graph in Figure S2B. In CF-T16AKO epithelia, under inflammatory conditions, the CPA-induced Isc was significantly less (p<0.01) compared to CF-CTRL epithelia (ΔIsc 1.8±1.8 µAmp*cm^{-2}, n=6, Figure S2B) and not different to the response of cells under normal conditions, indicating a non-TMEM16A resting activity.

Effect of forskolin in CF-CTRL and CF-26A4KO nasal epithelia.

FSK (10µM) was added bilaterally to CF-CTRL and CF-26A4KO epithelia, in the presence of amiloride (10µM). Under both normal and inflammatory conditions, the addition of FSK did not cause any acute change in Isc in both CF-CTRL and CF-26A4KO epithelia, as shown in the representative traces in Figure S3A. Interestingly, in both conditions, the measured drift Isc was smaller in CF-26A4KO epithelia, especially in the IL-4 treated epithelia (ΔIsc 0.8±0.6 µAmp* cm^{-2}, n=5, Figure S3B).

No evidence for SLC26A9 activity in CF nasal epithelia under normal and inflammatory conditions.

As shown in the representative traces in Figure S4A and B, GlyH-101 (10µM), a SLC26A9 inhibitor had no effect on Isc in the presence of amiloride (10µM), FSK (10µM), and the CFTR inhibitor 172 (20µM), from CF-CTRL and CF-26A9KO epithelia, for all the conditions tested.

No evidence for SLC26A4 protein expression in CF-26A4KO cells under normal and inflammatory condition.

As shown in the representative image in Figure S5A, SLC26A4 expression was not evident under normal conditions in both CF-CTRL and CF-26A4KO epithelia. However, following IL-4 treatment SLC26A4 was up-regulated in CF-CTRL epithelia and this effect was significantly reduced in CF-26A4KO epithelia, confirming the efficiency of the gene editing. Moreover, SLC26A4 colocalized with MUC5AC positive cells (goblet cells) as shown in the graph presented in Figure S5B.

SLC26A4 KO has no effect on forskolin-stimulated organoid swelling, but reduces organoid size under inflammatory conditions.

In order to evaluate if KO of SLC26A4 affected fluid secretion in nasal epithelia, a Forskolin Induced Assay (FIS) was performed in airway nasal organoids derived from the same CF donors. As shown in the representative images in Figure S6A the CF-26A4KO organoids developed similarly to the CF-CTRL under both normal and inflammatory conditions. As shown in Figure S6B the response to the agonist or the vehicle was similar in both CF-CTRL and CF-SL26A4KO under both normal and inflammatory conditions, as shown in the quantification of the AUC in Figure S6C. Under normal conditions, no significant differences between CF-CTRL and CF-26A4KO organoids were measured in the presence of FSK (1133.99±560.55 µm2, n=12, 1432.28±359.17 µm2, n=7, respectively). No significant differences were observed under inflammatory conditions between any of the conditions tested (1389.58±1450.05 µm2, n=12, 2043.56±950.65 µm2, n=7, respectively). However, CF-CTRL organoids presented a reduced initial lumen area under inflammatory condition that was significantly lower compared to the organoids under normal conditions (279.03±94.92 µm2, n=12 and 203.71±32.56 µm2, n=14, respectively, $p<0.05$, Figure S6D). Interestingly, in CF-26A4KO organoids the inflammatory conditions did not change the initial lumen size compared to normal conditions (314.04±86.48 µm2, n=5 and 298.70±60.25 µm2, n=5), and it was significantly different compared to CF-CTRL organoids ($p<0.05$, Figure S6D).

Fig. S1. Effect of carbachol on Isc in CF control and TMEM16A KO nasal epithelia under normal and inflammatory conditions.

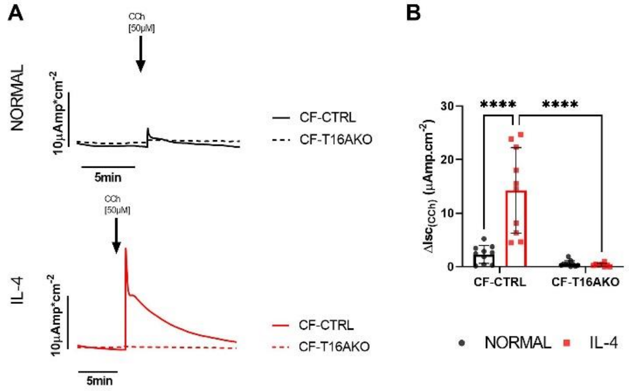

(A) Representative Isc traces after apical addition of carbachol (CCh, 50µM) in the presence of amiloride (10µM), from CF epithelia under normal (black traces) or inflammatory conditions (IL-4, 10ng/mL for 48 hrs, red traces). Solid traces, CF-CRISPRCas9 CTRL (CF-CTRL) and dotted traces, CF-TMEM16AKO (CF-T16AKO). **(B)** Summary of carbachol-induced change in Isc (ΔIsc CCh) (n=10, three donors, 2-way ANOVA with Tukey's multiple comparisons test). Data presented as Mean±SD, **** for $p<0.0001$.

Fig. S2. Cyclopiazonic acid (CPA) activates TMEM16A only in CF control nasal epithelia under inflammatory conditions.

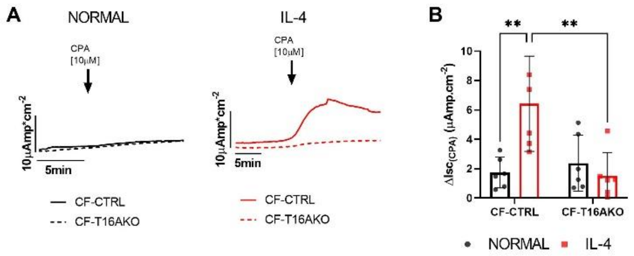

(A) Representative Isc traces after apical addition of cyclopiazonic acid (CPA, 10μM) in the presence of amiloride (10μM) from CF epithelia under normal (black traces) or inflammatory conditions (IL-4, 10ng/mL, for 48 hrs, red traces). Solid traces, CF-CRISPRCas9 CTRL (CF-CTRL) and dotted traces, CF-TMEM16AKO (CF-T16AKO). Left panel, normal conditions. Right panel; inflammatory conditions. **(B)** Summary of the CPA-induced change in Isc (ΔIsc CPA). (n=6, three donors, 2-way ANOVA with Tukey's multiple comparisons test). Data presented as Mean±SD, ** for $p<0.01$.

Fig. S3. Knock-out of SLC26A4 had no effect on Isc responses in CF control or CF-SLC26A4 KO nasal epithelia under normal and inflammatory conditions.

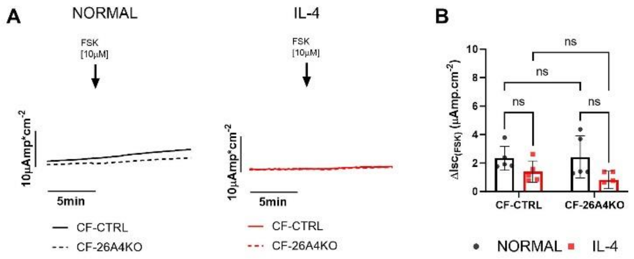

(A) Representative Isc traces after apical addition of forskolin (FSK, 10µM) in the presence of amiloride (10µM) from CF epithelia under normal (black traces) or inflammatory conditions (IL-4, 10ng/mL, for 48 hrs, red traces). Solid traces, CF-CRISPRCas9 CTRL (CF-CTRL) and dotted traces, CF-TMEM16AKO (CF-26A4KO). Left panel, normal conditions. Right panel; inflammatory conditions. **(B)** Summary of the forskolin-induced change in Isc (ΔIsc FSK). (n=5, three donors, 2-way ANOVA with Tukey's multiple comparisons test). Data presented as Mean±SD.

Fig. S4. No functional activity of SLC26A9 was detected by Isc or ASL pH measurements in CF nasal epithelia under normal and inflammatory conditions.

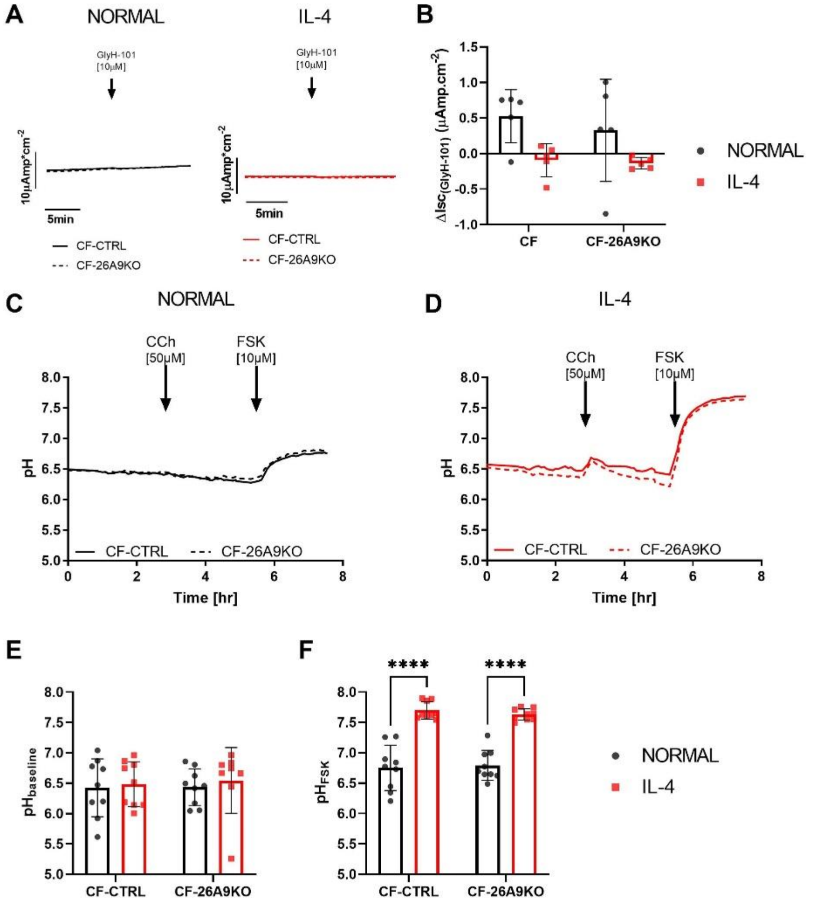

(A) Representative Isc traces after apical addition of GlyH-101 (GlyH-101, 10µM) in the presence of amiloride (10µM), forskolin (FSK, 10µM), and $CFTR_{inh}172$ (172, 20µM) from CF epithelia under normal (black traces) or inflammatory conditions (IL-4, 10ng/mL for 48 hrs, red traces). Solid traces, CF-CRISPRCas9 CTRL (CF-CTRL) and dotted traces, CF-SLC26A9 (CF-26A9KO). **(B)** Summary of GLyH-101-induced change in Isc (ΔIsc GlyH-101) (n=5, two donors). **(C)** Summary ASL pH traces. Solid black trace CF-CRISPRCas9 CTRL (CF-CTRL) and dotted black trace CF-SLC26A9KO epithelia (CF-26A9KO) under normal conditions. Baseline ASL pH was measured followed by the addition of carbachol (CCh, 50µM), and then forskolin (FSK, 10µM) as indicated. **(D)** Summary ASL pH traces. Solid red trace, CF-CRISPRCas9 CTRL (CF) and dotted red trace, CF-SLC26A9KO (CF-26A9KO) under inflammatory conditions (IL-4, 10ng/mL for 48hrs). Baseline ASL pH was measured followed by the addition of carbachol (CCh, 50µM), and then forskolin (FSK, 10µM) as indicated. **(E)** Baseline ASL pH. ASL pH was calculated as an average of five points before the addition of CCh. (n=9, two donors, 2-way ANOVA with Tukey's multiple comparisons test). **(F)** FSK ASL pH. ASL pH was calculated as an average of five points after two hours of the addition of FSK. (n=9, two donors, 2-way ANOVA with Tukey's multiple comparisons test). Data presented as Mean±SD, ** for p<0.01, **** for p<0.0001.

Fig. S5. Immunofluorescent validation of SLC26A4 confirmed its expression only in CF-CTRL nasal epithelia under inflammatory conditions.

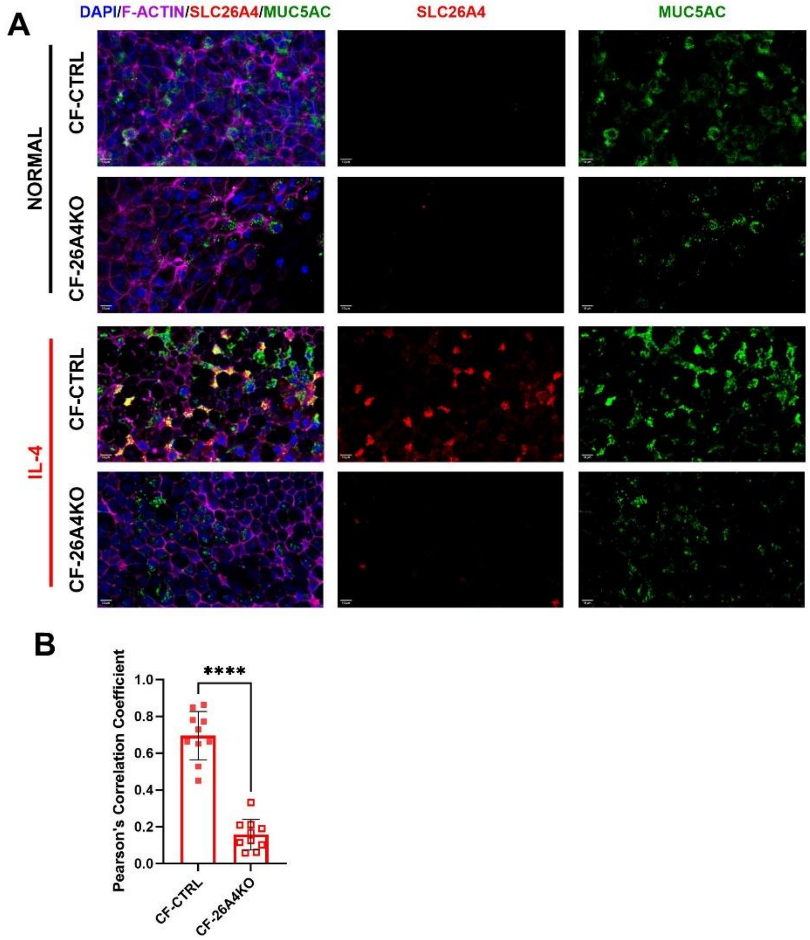

(A) Representative immunofluorescent (IF) images of CF-CTRL and CF-26A4KO epithelia under normal (black line) and inflammatory conditions (IL-4, 10ng/mL for 48 hrs, red line). Epithelia were stained using antibodies against SLC26A4 (red), MUC5AC (green) and F-actin (magenta). Scale bar 10µm. **(B)** Summary of colocalization between positive signals of SLC26A4 and MUC5AC in CF-CTRL and CF-26A4KO epithelia under inflammatory conditions (n=10, one donor, unpaired t-test). Data presented as Mean±SD, **** for p<0.0001.

Fig. S6. Knock-out of SLC26A4 had no effect on cAMP stimulated fluid secretion but did reduce mean organoid size in CF nasal airway organoids under inflammatory conditions.

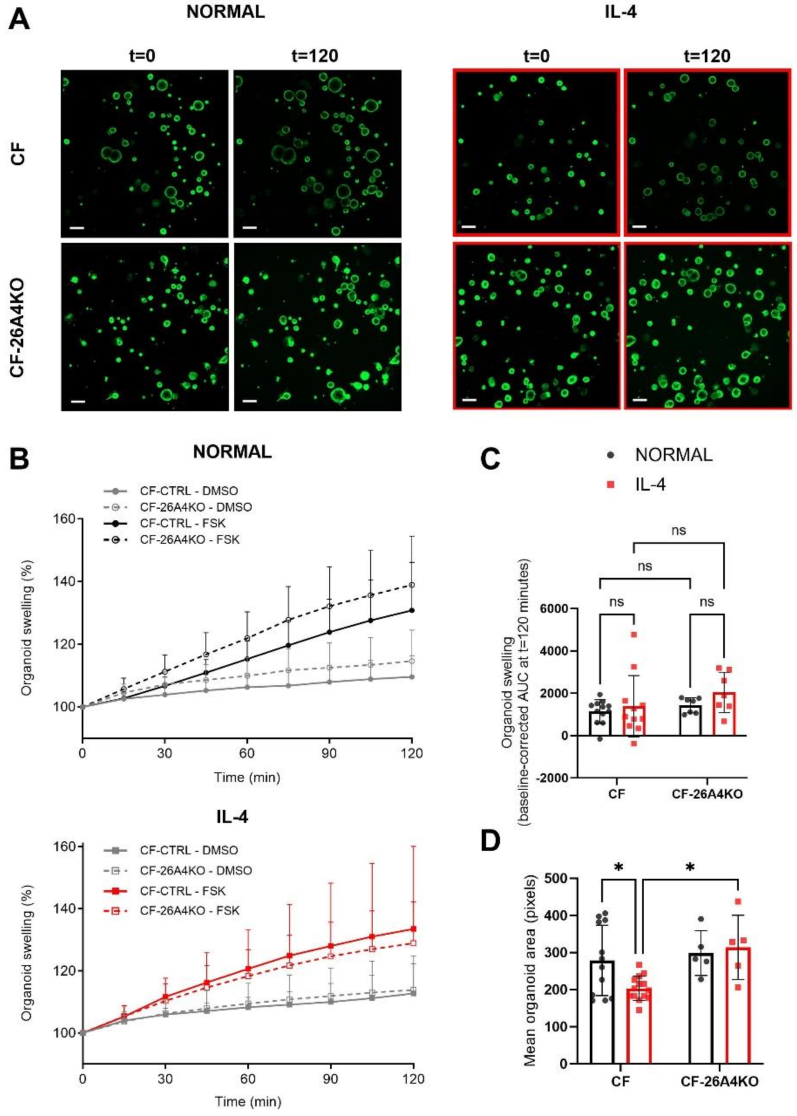

(A) Representative confocal images of CF-CRISPRCas9 CTRL (CF-CTRL) and CF-SLC26A4KO (CF-26A4KO) nasal organoids, stimulated with forskolin (FSK, 10 µM) at 0 and 120 min under normal and inflammatory conditions (IL-4, 10ng/mL, for 48 hrs). Scale bar 200µm; **(B)** Summary of the forskolin-induced fluid secretion in nasal organoid swelling. Top panel, normal conditions. In solid black traces CF-CTRL treated with FSK, and in solid gray traces CF-CTRL treated with DMSO, and in dotted black traces CF-26A4KO treated with FSK, and in dotted gray traces CF-26A4KO treated with DMSO. Bottom panel; inflammatory conditions. In solid red traces CF-CTRL treated with FSK, and in solid gray traces CF-CTRL treated with DMSO, and in dotted red traces CF-26A4KO treated with FSK, and in dotted gray traces CF-26A4KO treated with DMSO. **(C)** Summary of the Area Under the Curve (AUC) of the forskolin-induced swelling. AUC corrected by subtracting the DMSO control values. (n=7-12, three donors, 2-way ANOVA with Tukey's multiple comparisons test). **(D)** Summary of the mean area of the organoids at t=0 under normal and

inflammatory conditions (n=7-14, three donors, 2-way ANOVA with Tukey's multiple comparisons test). Data presented as Mean±SD, * for $p<0.05$.

Fig. S7. CRISPR-Cas9 gene editing efficiency.

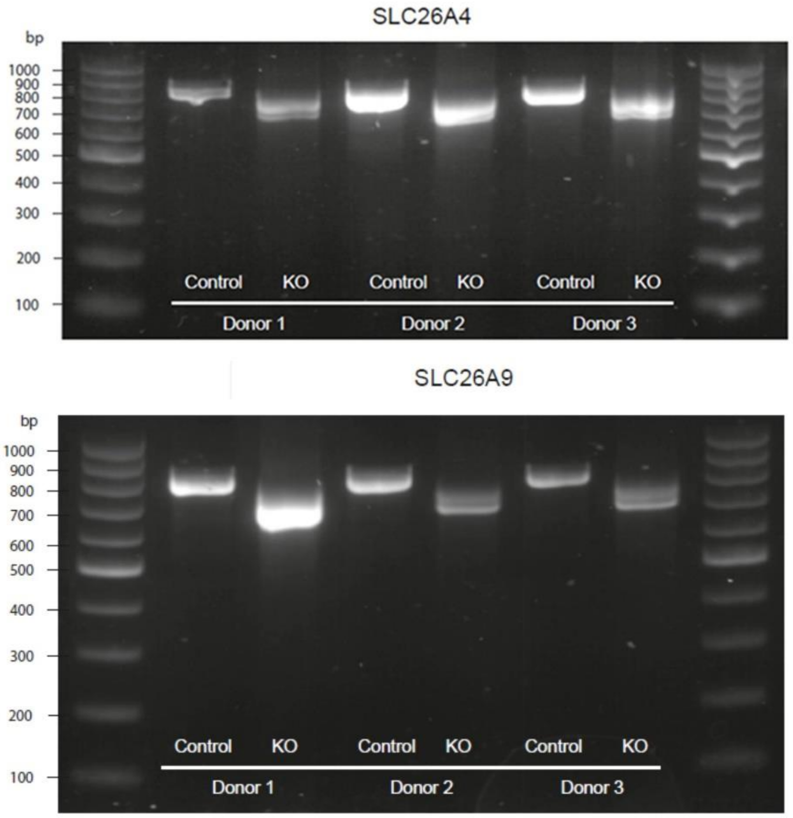

	G542X/CFTRdele2.3 (21 kb)	W1282X/1717-1G>A	R553X/R553X
SLC26A4	86	86	88
SLC26A9	85	86	77

DNA gel showing the PCR-amplified products of the targeted SLC26A4 and SLC26A9 genes for KO and control samples of 3 CFTR-null donors (G542X/CFTRdele2.3 (21 kb), W1282X/1717-1G>A, R553X/R553X). The predicted length of the PCR product was 835 bp for CF-26A4KO and 823 bp for CF-26A9KO. The table indicates the percentage KO efficiency.

Supporting Materials and Methods

Cell culture.

Nasal brushings were collected from donors after obtaining their informed consent. All biobank participants gave written, broad informed consent for long term biobanking and future research activities of their airway cells generated from nasal brushings, as approved by the Medical Research Ethics Committee of the University Medical Center Utrecht (TcBIO protocol ID: 16/586). Participants had adequate time for deciding to participate, were not included by their treating physician, and always retained the right to withdraw materials from the biobank. The specific use of cells from this biobank for all of the research activities described in this manuscript complied with the original informed consent of all of the biobank participants and the full study protocol was approved by the ethical committee under protocol TcBIO ID: 19/764). Briefly, basal cells were isolated and expanded in T75 flasks using feeder-free, and growth factor-defined culture conditions. Once cells reached 80% confluency, they were seeded at a density of 0.2*106 cells onto 6.5mm porous supports (0.4µm pore size, 3470 Corning) pre-coated with human collagen IV (C7521, Sigma) and cultured under liquid-liquid interface with expansion medium until confluent. The cells were then fully differentiated for 28 days using the air-liquid interface (ALI) method, which was changed three times/week using the ALI differentiation medium. The day before experiments, inserts were washed with sterile PBS (14190-144, Thermo Fisher) for 10 mins at 37°C, 5% CO_2 and the ALI medium was changed. To induce a pro-inflammatory phenotype, CF epithelia were pre-treated with IL-4 (10ng/mL, 200- 04, Peprotech, London, UK) for 48 hours before experiments.

Knock-out of TMEM16A, SLC26A4, and SLC26A9 using CRISPR-Cas9.

Briefly, 1 million nasal epithelial cells were mixed with multi-guide sgRNA (30 µM; Synthego, Redwood City, CA, USA) and recombinant 2NLS-Cas9 nuclease (20 µM, Synthego, Redwood City, CA, USA) and electroporated in bulk using a NEPA21 electroporator (Nepa Gene, Ichikawa City, Japan). After electroporation, cells were expanded and differentiated as described above. Gene editing efficiency was quantified by amplifying regions of interest with GoTaq G2 Flexi DNA polymerase (Promega, Madison, WI, USA). Amplified DNA fragments were excised from a 1.2% TBE-agarose gel, purified according to the Gel Extraction Kit (Qiagen, Venlo, The Netherlands) and sent for Sanger sequencing. KO efficiency was analysed with the ICE analysis tool (www.ice.synthego.com, accessed on 21 January 2021).

ASL pH measurements

Briefly, the ASL was stained with 3 µl of a mixture of dextran-coupled pH-sensitive pHrodo Red (0.5 mg/mL, λex: 565 nm, λem: 585 nm, P10361, Thermo Fisher) and Alexa Fluor® 488 (0.5 mg/mL, λex: 495 nm, λem: 519 nm, D22910, Thermo Fisher) diluted in glucose-free HCO_3^-KRB, overnight at 37°C, 5% CO_2. During the experiments, all chemicals were added to the basolateral bathing solution. pHrodo and Alexa Fluor® 488 fluorescence was measured every 5 mins and data was analyzed by subtracting the background values from each time point, and then a ratio from pHrodo and Alexa Fluor® 488 was calculated. Finally, pH values were obtained by interpolating the calculated ratio with a standard curve performed at the end of each experiment, using buffered solutions of fixed pH (5.5, 7.0, 8.0). Changes in ASL pH (ΔASLpH) were calculated by averaging 5 time points before and 5 time points after the addition of chemicals as described in each figure legend.

Short-Circuit Current Measurements

Monolayers were submerged with bilateral HCO_3^-KRB solution continuously gassed with 95% O_2-5% CO_2 and maintained at 37°C, voltage-clamped to 0mV and left to equilibrate for 30 min before the addition of chemicals. These were added in the following sequence: amiloride (amil, 10 µM, apical; followed by carbamylcholine chloride (CCh, 50 µM, basolateral), or cyclopiazonic Acid

(CPA, 10 µM, basolateral), or forskolin (Fsk, 10 µM, bilateral). The transepithelial short-circuit current (Isc) was recorded every 10-s using Ag-AgCl electrodes in 3M KCl agar bridges, and results normalized to an area of 1 cm^2 and expressed as $\mu Amp*cm^{-2}$ using the Acquire & Analyze software (Physiologic Instruments). Changes in short-circuit current (ΔIsc) were then calculated by averaging 5 time points before and 5 points after the addition of chemicals.

Immunofluorescence staining and protein colocalisation

HNEC epithelia were fixed with PFA, washed with PBS at RT with gentle shaking before being permeabilized with 0.3% Triton in PBS, blocked in 5% BSA in PBS (1 h, RT), washed in PBS, and incubated over night at 4 C with the primary antibodies against MUC5AC (1:67, Abcam ab3649) and SLC26A4 (1:100, Thermo Fisher Scientific PA5-115911) diluted in 1% BSA in PBS. The epithelia were then washed 3 times in PBS and incubated in the dark with the secondary antibodies goat anti-rabbit (5 µg/mL, Thermo Fisher Scientific A11034) and goat anti-mouse (2 µg/mL, Thermo Fisher Scientific A11032) together with Phalloidin-Alexa Fluor 647 (A30107, Thermo Fisher Scientific) diluted in 1% BSA in PBS, for 1 h at room temperature. DAPI (in PBS) was added for 5 min before membranes were further washed (5 min, PBS). The membranes with the epithelia were placed on slides (10149870, Thermo Fisher Scientific), and mounting medium added (H-1000-10, VECTASHIELD, Vector laboratories) coverslips were added and closed using transparent nail polish. Images were captured as z stacks using a Leica SP8 confocal microscope equipped with a 63x/1.4 PlanApo objective (Leica, Germany) with Nyquist sampling in xy and z. Channels were captured sequentially to avoid crosstalk and the data were then deconvolved and colocalisation analysis performed in Huygens (v 23.04, SVI, Netherlands). Colocalisation analysis was performed between Alexa488 and Alexa594 channels with Coste's method of background estimation set on one field of data and re-used for others. Pearson correlation co-efficients were obtained for individual cells using the ROI tool. Data were plotted in R (v 4.2.1) and statistical tests performed in LibreOffice (v 7.4). Representative images were taken using the Fiji software (1).

Generation of Airway Organoids and Organoids swelling assay

Differentiated ALI HNEC epithelia, were used to generate airway organoids (AO) as previously described (2, 3). Briefly, to generate AO the ALI epithelia were washed with DPBS and the basolateral medium was changed with a fresh ALI medium. To the basolateral medium was replaced with advanced DMEM/F12 medium (11550446, Thermo Fisher Scientific) supplemented with 1mg/mL collagenase type II (10738473, Thermo Fisher Scientific) and incubated at 37°C with 5% CO2 for 45-60 min. The detached epithelia were collected with fresh advanced DMEM/F12 medium supplemented with 10% FBS to stop collagenase activity added on the apical side to enable the collection of the epithelia sheet, which was transferred into a conical tube. The epithelial sheet was then disrupted in small fragments (maximum size ~100 µm) using mechanical pipetting, and the cell suspension was strained into a new conical tube using a 100 µm strainer (431752, Corning). The sheets were centrifuged for 5 min at 1200 rpm at 4°C. The pellet was then resuspended in Matrigel (354230, Corning) diluted 1:4 in AO medium and then 30µL droplets were plated into each well of a pre-warmed 24 well plate (3524, Corning). The Matrigel droplets were solidified placed upside down at 37°C for 15-30 min. Once the droplets were solidified, AO medium was added. The plate was incubated at 37°C and the medium changed every 48hrs. The organoid structure developed after 24 hours, and the cultures were ready to be used after 4 days. After 4 days, when the organoid structure is fully developed, the droplets were disrupted to collect the organoids. The medium was removed and replaced with an equal volume of cold Cell Recovery Solution (354253, Corning) and placed at 4°C for 30 min to dissolve the Matrigel. Once the Matrigel was completely liquified the organoids were collected in a centrifuge tube and the wells were washed with cold advanced DMEM/F12 medium. The organoids were centrifuged for 5 min at 1200 rpm at 4°C. The organoids pellet was resuspended in Matrigel (354230, Corning) diluted 1:2 in AO medium and then 4µL droplet, containing 25-50

organoids, were plated into each well of a pre-warmed 96-well Flat Clear Bottom Black microplates (3603, Corning), the experiments were performed 48 hours after plating. To induce a pro-inflammatory phenotype, CF epithelia were pre-treated with IL-4 (10ng/mL, 200- 04, Peprotech, London, UK) for 48 hours before experiments.

For the airway organoid swelling assay, the 96-well plates containing the organoids were stained for 30 min at 37°C with 3µM Calcein green AM (λex 495 nm, λem 515 nm; 10462052, Thermo Fisher Scientific), dissolved in DMSO. The assay was performed with a Zeiss Axio Observer Z1/7 inverted microscope using a Fluor 5x/0.25 objective; an LSM800 detector for fluorescence and ESID detector for transmitted light were used; the two channels were recorded sequentially with bidirectional scanning. The whole plate was first scanned to obtain the time 0 image and then forskolin (10µM in DMSO) or DMSO were added to each well. One image per well was collected every 15 min for 2 hours (n=9). For the entire experiment, the organoids were kept at 37°C with 5% CO2. The fluorescent images were analysed using the Zeiss proprietary software ZEN 2.5 (blue edition). To measure the swelling of the AOs, the average change in the surface area of calcein-treated AOs was calculated over time per well. The average area calculated on the first image (time 0) for each well was set to 100%. The following time course images were then normalized to the time 0 area, and then the percentage surface area of calcein-stained AOs was obtained. Area Under the Curve (AUC) values were calculated from the percentage of swelling calculated. The AUC values from DMSO treated organoids were subtracted from the AUC of FSK-treated organoids from the same experiment.

References

1. J. Schindelin *et al.*, Fiji: an open-source platform for biological-image analysis. *Nat Methods* **9**, 676-682 (2012).
2. G. D. Amatngalim *et al.*, Measuring cystic fibrosis drug responses in organoids derived from 2D differentiated nasal epithelia. *Life Sci Alliance* **5** (2022).
3. L. W. Rodenburg *et al.*, Drug Repurposing for Cystic Fibrosis: Identification of Drugs That Induce CFTR-Independent Fluid Secretion in Nasal Organoids. *International Journal of Molecular Sciences* **23**, 12657 (2022).
